# Supplementary material for: Glutathione S-Transferase May Contribute to the Detoxification of (S)-(−)-Palasonin in Plutella xylostella (L.) via Direct Metabolism
Source: Insects. 2022 Oct 28;13(11):989. doi: 10.3390/insects13110989 (PMC9692725; doi:10.3390/insects13110989)
Supplement: Supplementary file 1 [file insects-13-00989-s001.zip › insects-1968948-supplementary.pdf]

Supplementary Tables

Table S1. Primer sequences

Gene	Primer	Sequence(5'-3')
GSTd1	Forward primer	TGGAAATGGTGCTGACAAAC
	Reverse primer	GGGTTCTTGGGGTAGAGAGC
GSTd2	Forward primer	CTCAACCCTCAACACACGGT
	Reverse primer	ACCGTGTGTTGAGGGTTGAG
GSTs1	Forward primer	AAGAACTACCCGCACGTGAA
	Reverse primer	TAGCGCGTAGACCTTCCTCT
GSTs2	Forward primer	AAGCCCAAGACCAAGTACGG
	Reverse primer	ATCTCGAAGTTCTGCTCCGC
β-actin	*Forward primer*	*GAGGTATCCTCACCCTGAAGTA*
	Reverse primer	*ACGCAGCTCGTTGTAGAAG*

Supplementary Figures

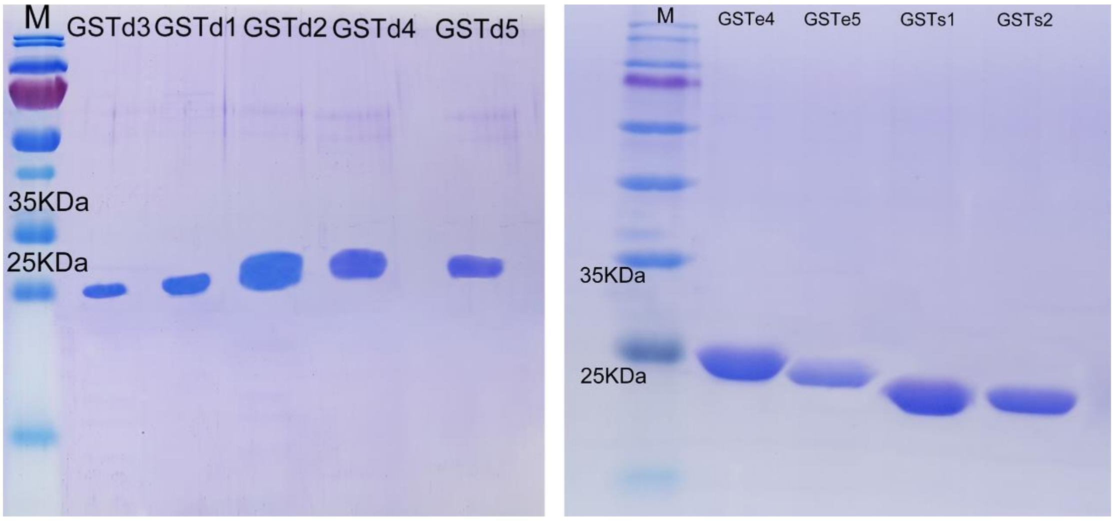

Figure S1. The purified GSTs recombinant protein was analyzed by 15 % SDS-PAGE.

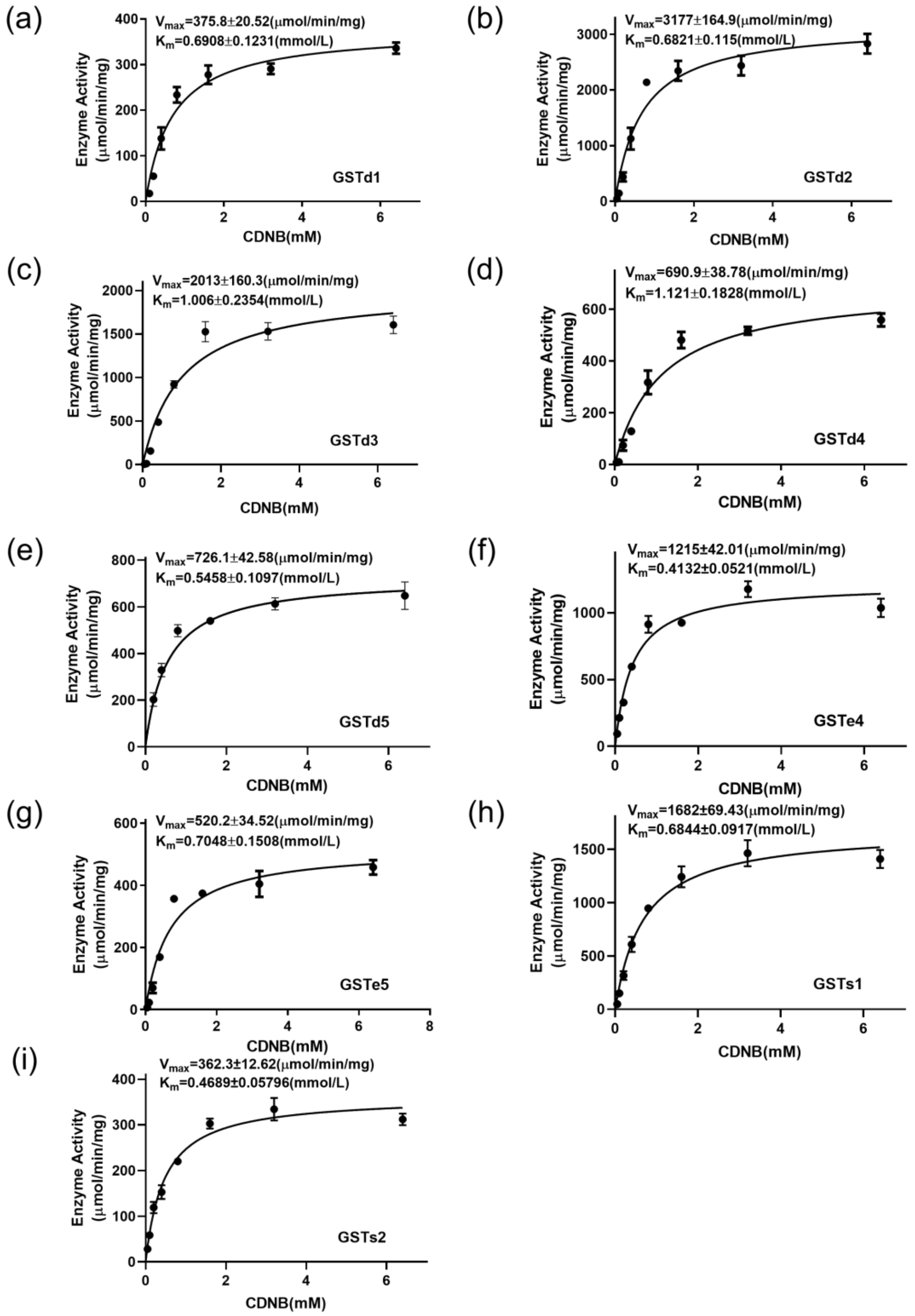

Figure S2. Enzyme kinetics of recombinant GSTs proteins. GSTd1 (a), GSTd2 (b), GSTd3 (c), GSTd4 (d), GSTd5 (e), GSTe4 (f), GSTe5 (g), GSTs1 (h) and GSTs2 (i)
